# Enhanced Energetic Performances Based on Integration with the Al/PTFE Nanolaminates

**DOI:** 10.1186/s11671-018-2618-y

**Published:** 2018-07-11

**Authors:** Yuxin Zhang, Yichao Yan, Yao Wang, Mengting Ai, Hongchuan Jiang, Liang Wang, Xiaohui Zhao, Wanli Zhang, Yanrong Li

**Affiliations:** 1 State Key Laboratory of Electronic Thin Films and Integrated Devices, University of Electronic Science and Technology of China, Chengdu, 611731 China; 2 Institute of Chemical Materials, China Academy of Engineering Physics, Mianyang, 621999 China

**Keywords:** Al/PTFE nanolaminates, Nanostructured energetic materials, Exploding foil initiator, Electric initiation

## Abstract

Integrating energetic materials on a chip has received great attention for its widely potential applications in the microscale energy consumption system, including electric initiation device. In this article, reactive Al/PTFE nanolaminates with periodic layer structure are prepared by magnetron sputtering, which consists of fuel Al, oxidant PTFE, and inert layer Al-F compound in a metastable system. The as-deposited Al/PTFE nanolaminates exhibit a significantly high energy output, and the onset temperature and the heat of reaction are 410 °C and 3034 J/g, respectively. Based on these properties, an integrated film bridge is designed and fabricated via integrating Al/PTFE nanolaminates with a Cu exploding foil, which exhibits enhanced energetic performances with more violent explosion phenomenon, larger quantities of ejected product, and higher plasma temperature in comparison with the Cu film bridge. The kinetic energy of flyers derived from the expansion of the Cu film bridge is also increased around 29.9% via integration with the Al/PTFE nanolaminates. Overall, the energetic performances can be improved substantially through a combination of the chemical reaction of Al/PTFE nanolaminates with the electric explosion of the Cu film bridge.

## Background

Over the last decade, investigations into nanostructured energetic materials have received worldwide concern and increasing research interest owing to their superior energetic performances, including low ignition temperature, rapid energy release, high energy density and tunable reactivity [1–10]. The chemical energy stored by these materials can be released upon electrical, optical, impact, or thermal actuation, which can be used for military purposes and civilian applications, such as initiation of secondary reactions [11], joining of materials [12], automotive air-bag propellants [13], and power supply [14]. Many methods including the physical mixing of nanopowders, arrested reactive milling of dense nanocomposites, electrophoretic nanoenergetic coating, and periodical deposition of nanolaminates have been introduced to fabricate nanostructured energetic materials [15–19]. Among these methods, the fabrication of nanolaminates through alternately depositing two or more different films provides a fascinating structure for device integration with tunable energetic performances, because the number of layers and the thickness of monolayer are easily controlled, and consequently to tune their energetic performances.

Exploding foil initiator (EFI) is a type of electric generation pyrotechnic devices used for initiation of secondary reactions [20]. After applying an electric pulse, instantly increasing current density causes the vaporization of metal film bridge and the generation of high pressure plasma. Then, the flyer on the film bridge is sheared and accelerated to impact the explosives. With the increasing requirements for electric ignition device miniaturization and low energy initiation, the integration of nanoenergetic layers with a metal film bridge based on microelectronic and mechanical system (MEMS) technology to achieve functional nanoenergetic-on-a-chip (NOC) constitutes a promising option for the development of EFI. The combination of the reaction heat of energetic materials with the traditional electrical joule of metal film bridge make it possible to improve the electric explosion performances of EFI with low energy initiation in a compact size.

Al/PTFE nanolaminate film is a promising candidate to be integrated with EFI based on the following reasons. First, the metal Al is a common material with a high energy density and energy release rate during oxidation. Meanwhile the fluorine content in PTFE is up to 76 wt.%, which can react with the metal Al to form AlF_3_ with a high theoretical energy release of 5571 J/g [21]. Second, the potential gas release derived from the pyrolysis of PTFE film and the reaction product of oxycarbide in the atmospheric conditions can increase the pressure of generated plasma, which is beneficial for shearing and accelerating of the flyer [22]. In this paper, an integrated film bridge was designed and fabricated via integrating the Al/PTFE nanolaminates with a Cu exploding film bridge. The structure and chemical composition of as-deposited Al/PTFE nanolaminates were studied by TEM and XPS analyses. The effects of the integrated Al/PTFE nanolaminates on the electric initiation performances were investigated through the electric explosion tests.

## Methods

### Deposition of the Al/PTFE Nanolaminates

Al/PTFE nanolaminates were prepared through alternately depositing Al layers and PTFE layers by direct current magnetron sputtering and radio frequency magnetron sputtering, respectively. The targets used for sputtering were pure aluminum foil (purity > 99.999%) and polytetrafluoroethylene foil (purity > 99.99%) with a size of 100 mm in diameter. A rotating substrate table was employed to realize multiple alternating depositions. The base pressure for film deposition was below 5 × 10^− 4^ Pa, and the argon gas was introduced as gas media. The deposition parameters were set as 1.1 Pa, 300 W for PTFE layers, and 0.45 Pa, 100 W for Al layers, to obtain an optimized film quality and stable deposition rate.

### Preparation of the (Al/PTFE)_n_/Cu-Integrated EFI

**Fig. 1:**
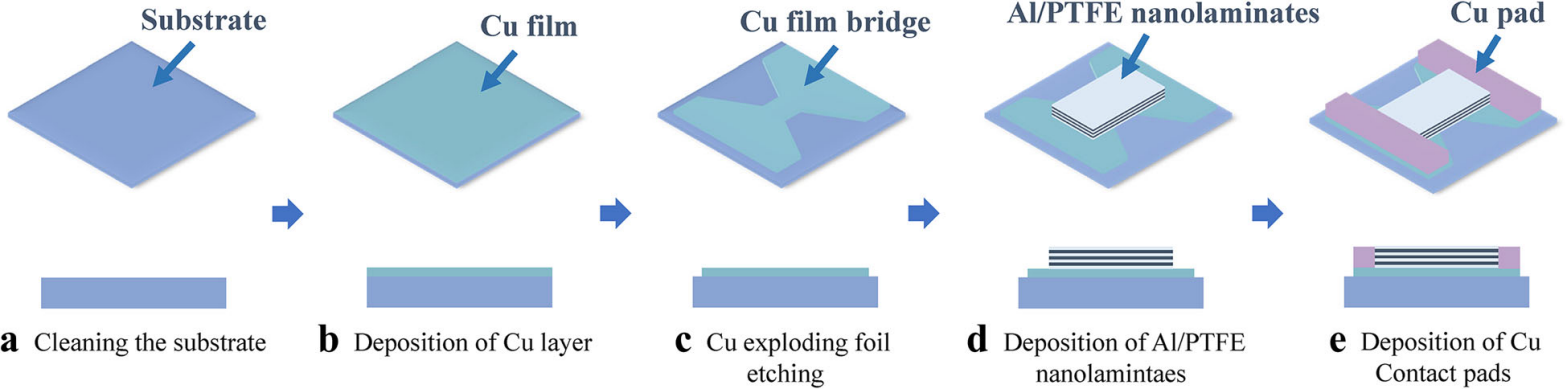
Schematic drawing and fabrication process flow of the (Al/PTFE)_n_/Cu film bridge

The (Al/PTFE)_n_/Cu film bridge was prepared by magnetron sputtering and MEMS techniques on an alumina ceramic substrate with a diameter of 3 in.. The fabrication process of the (Al/PTFE)_n_/Cu film bridge is shown in Fig. 1. Each unit consists of a Cu exploding film bridge on the bottom, a rectangular shape Al/PTFE nanolaminate film deposited on the top of a Cu film bridge, and two lands of Cu pads located at the both sides of the Al/PTFE nanolaminates.

Before deposition, the substrate was ultrasonic-cleaned sequentially by using acetone, alcohol, and deionized water for 10 min. Next, the cleaned substrate was blow-dried by argon gas and heat treated at 120 °C for 1 h for further drying. After drying, a 2-μm-thick Cu layer was deposited on the cleaned substrate by DC magnetron sputtering. Subsequently, the as-deposited Cu film was patterned through photolithography, and wet-etched by copper etching agent (CE – 100). The dimension of the patterned Cu film bridge was 600 μm × 600 μm. Then, ~ 2-μm-thick Al/PTFE nanolaminates were deposited on the top of the Cu film bridge and patterned with image reversal lift-off process. The stacking sequence for sputtering Al/PTFE nanolaminates was Al/PTFE/Al/PTFE/Al, and Al layer was left as the top layer. After that, two Cu contact pads patterned with mask were stacked on the both sides of the Al/PTFE nanolaminates for the connection to the voltage source. Finally, the finished sample was diced into individual units.

### Characterization of the Al/PTFE Nanolaminates

The crystallinity and structural microscopic characterization of the Al/PTFE nanolaminates were performed using transmission electron microscopy (TEM). A ~ 1-nm-thick Al film was deposited on the PTFE layer to determine the chemical compositions of the interface between Al layer and PTFE layer by X-ray photoelectron spectroscopy (XPS). The PTFE nanolaminates were scrapped from the substrate and transferred in an alumina crucible for the analysis of energy release by differential scanning calorimetry (DSC). The sample mass for each test was ~ 10 mg, and the tests were carried out from 25 to 800 °C at a heating rate of 10 °C/min in flowing argon.

### Electric Explosion Test of the Film Bridge

The electric explosion properties of the samples were tested by an electric explosion measurement system, which is similar to the previous report for Cu/Al/CuO film bridge [23]. The electric explosion temperature characteristics were determined by an electric explosion temperature diagnosis mode based on the “double-line atomic emission spectroscopy of a copper element” [24, 25]. The electric explosion phenomena were recorded synchronously by a high-speed camera with 20,000 frames per second. The acceleration process of flyers was obtained through photonic Doppler velocimetry (PDV) to investigate the ability to drive flyers.

## Results and Discussion

### Characterization of the Al/PTFE Nanolaminates

**Fig. 2:**
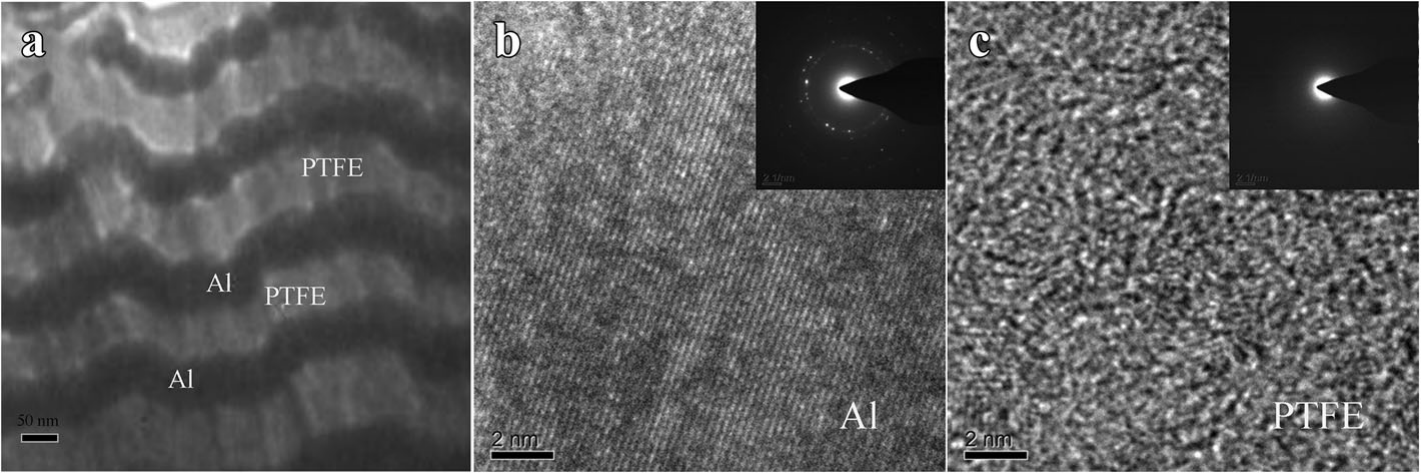
**a** Cross-sectional bright-field TEM image of the Al/PTFE nanolaminates. **b** High-resolution image of the Al layer and the electron diffraction pattern is inserted. **c** High-resolution image of the PTFE layer and the electron diffraction pattern is inserted

The cross-sectional TEM image of the Al/PTFE nanolaminates is shown in Fig. 2a. The Al layers and PTFE layers are arranged periodically in vertical orientation, and the well-aligned layer structure is clearly visible. The dark strips correspond to the Al layers, while the bright strips match the PTFE layers. The Al layers and PTFE layers can be distinguished easily, and the wavy interfaces between Al layers and PTFE layers are also visible in the image. The monolayer thickness of the Al layer and PTFE layer are about 50 and 75 nm, respectively. The high-resolution images of the Al layer and PTFE layer are shown in Fig. 2b, c, and the electron diffraction patterns are inserted. The lattice arrangement of the Al film can be observed clearly, which exhibits a well-defined nano-polycrystalline structure. While the PTFE film exhibits broad and diffuse rings, indicating amorphous structure. The periodic layer structure is beneficial for interfacial diffusion between Al layers and PTFE layers to release energy. The homogeneous film thickness also enables the tunable energetic performances by changing the thickness of each layer and the number of layers.

**Fig. 3:**
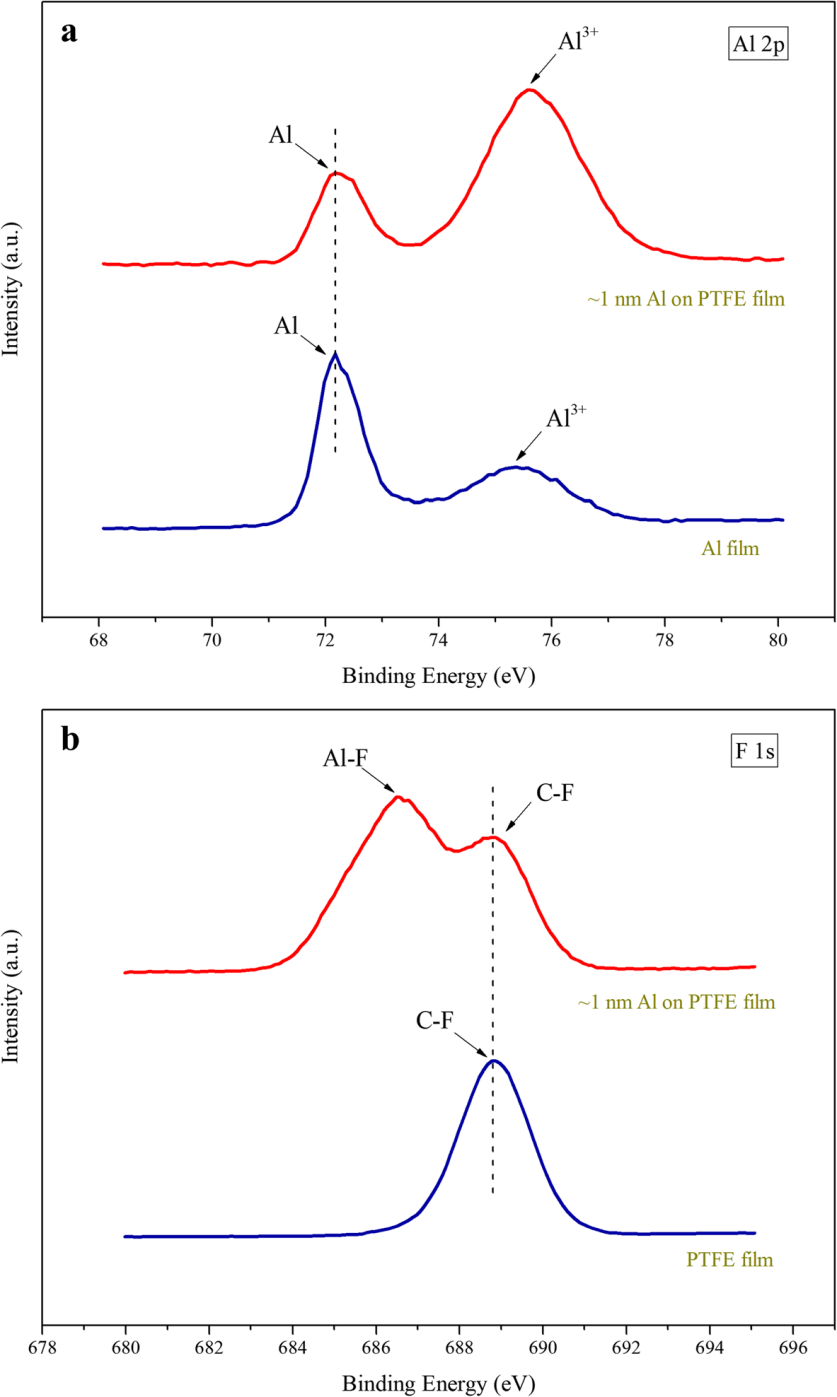
**a** High-resolution spectrum of Al 2p core level of the Al film and PTFE film with a ~ 1-nm-thick Al overlayer. **b** High-resolution spectrum of F 1s core level of the PTFE film and the PTFE film with a ~ 1-nm-thick Al overlayer

To further confirm the interfacial chemical composition between Al layer and PTFE layer, XPS analyses are performed on the samples of Al film, PTFE film, and PTFE film with a ~ 1-nm-thick Al layer deposited on the surface. Figure 3a shows Al 2p core level spectra of the Al film and PTFE film with deposition of ~ 1-nm-thick Al. The peaks of Al 2p core level which appeared at binding energy (BE) of 72.2 eV are due to the metallic Al. The peaks at 75.4 eV from Al film, and 75.6 eV from ~ 1 nm Al deposited on PTFE film could be attributed to the oxidized aluminum. Compared to the PTFE film without Al deposited on surface, the peak of Al 2p core level that match Al^3+^ shift slightly to higher binding energy. It might be induced by the reaction between Al and PTFE [26, 27]. Meanwhile, Fig. 3b shows the changes in F 1s core level of the PTFE film before and after the deposition of ~ 1 nm Al. The peak at 686.6 eV fits well with Al-F bonds in AlF_3_, which clearly demonstrates that the chemical reaction occurs at the interface between the Al layer and PTFE layer at the initial stage of film deposition. These results also prove that the Al/PTFE nanolaminates are in a metastable reaction system consisting of fuel Al, oxidant PTFE, and inert layer Al-F compound. Small amounts of Al-F bonds which exist at the interfaces of Al/PTFE nanolaminates could prevent the continuous reaction between PTFE and Al, which are important components to keep high energy density and stability of the Al/PTFE nanolaminates [28].

**Fig. 4:**
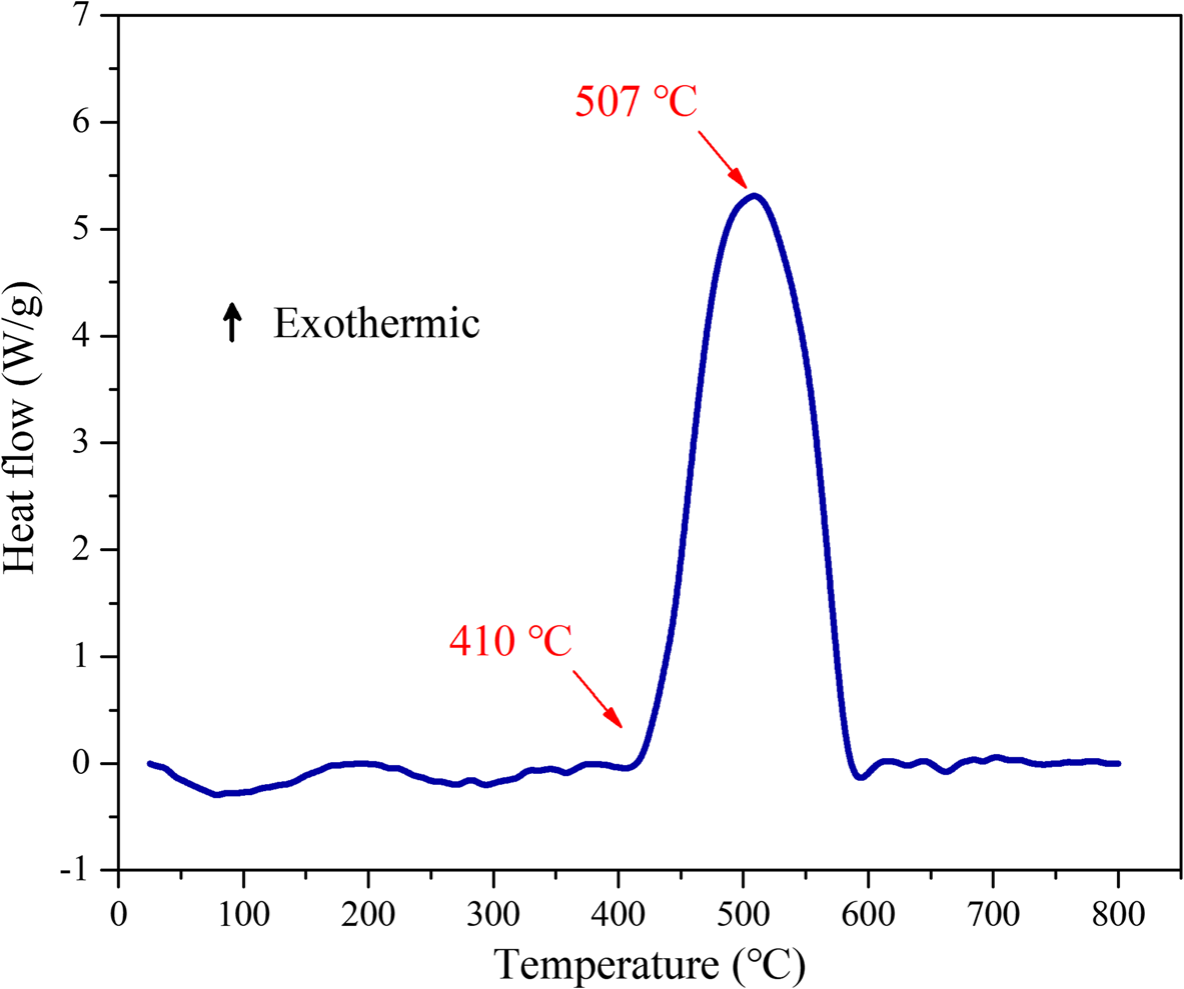
DSC curves of the Al/PTFE nanolaminates as a function of temperature in argon environment

The heat release characteristics of the Al/PTFE nanolaminates were tested by DSC in a temperature range of 25 to 800 °C under constant heating rate of 10 °C/min in flowing argon. As shown in Fig. 4, a major exothermic peak is observed to rise abruptly at the temperature value of 507 °C, which is associated with the oxidation-reduction reaction between Al and PTFE. The onset reaction temperature of the Al/PTFE nanolaminates is 410 °C, and the heat of reaction is about 3034 J/g calculated by integrating the positive exothermic heat flow with respect to time. The Al/PTFE nanolaminates exhibit significantly high energy output with a relatively low onset reaction temperature. Note that the heat of reaction is below the maximum theoretical value; this might be caused by the reactions which are incomplete during the temperature rising, and the formation of Al-F compound layer at the interfaces decreases the heat release slightly.

### Electric Initiation Performances of the (Al/PTFE)_n_/Cu Film Bridges

**Fig. 5:**
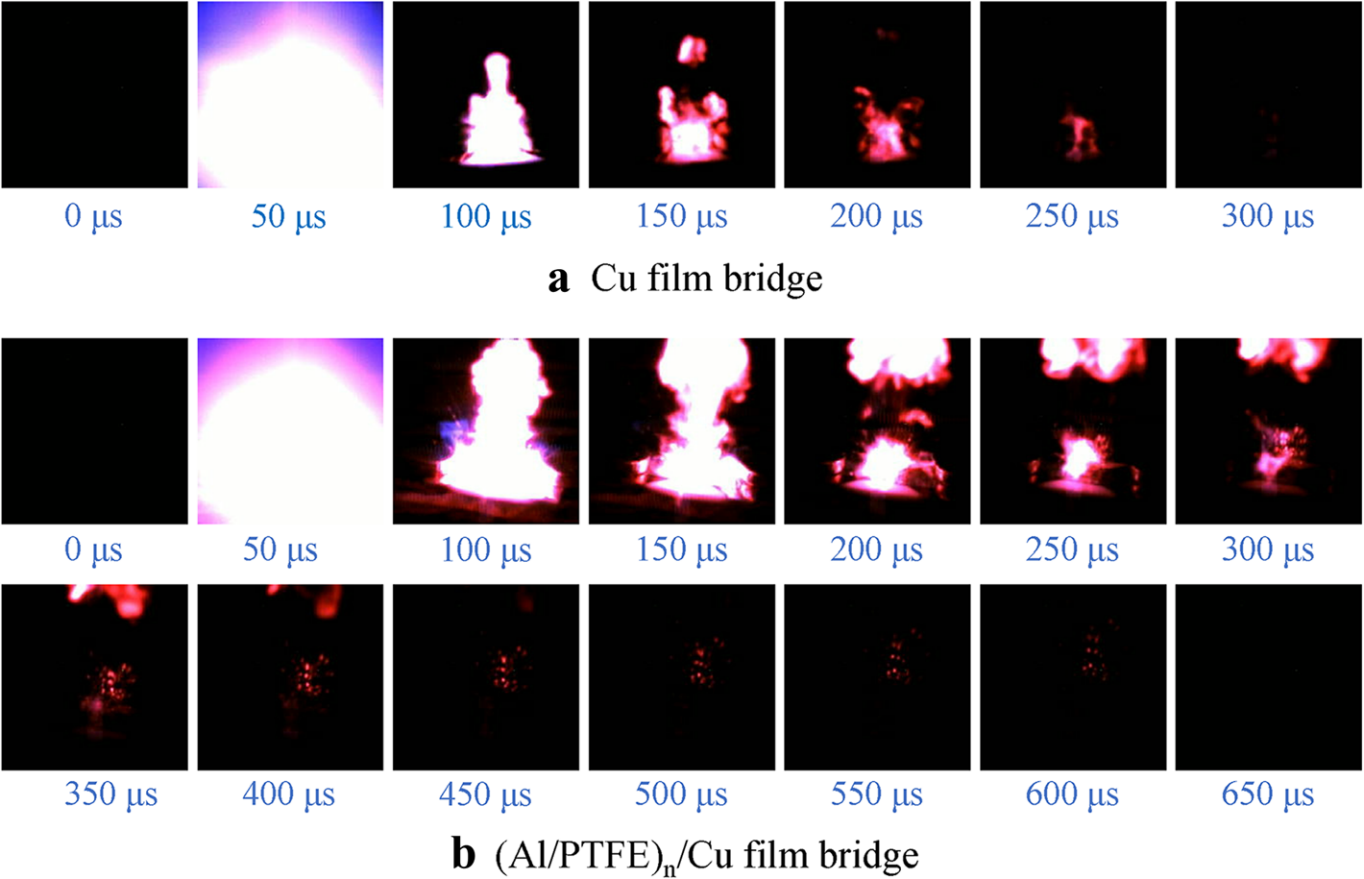
High-speed camera observation of the electric explosion processes for the Cu film bridge (**a**) and the (Al/PTFE)_n_/Cu film bridge (**b**) at a 2500-V discharge voltage

Based on the structure and exothermic properties of the Al/PTFE nanolaminates, an integrated film bridge was fabricated by integrating Al/PTFE nanolaminates with a Cu exploding film bridge. A sequence of high-speed video frames for the electric initiation phenomenon of the Cu film bridge and the (Al/PTFE)_n_/Cu film bridge were recorded at a 2500-V discharge voltage, as shown in Fig. 5; the interval between adjoining pictures is 50 μs. After discharging the stored electrical energy through the bridge, a violent electric explosion process accompanied with a bright flash is observed on the Cu film bridge. This indicates a rapid state change from solid to ionized plasma which occurred on the Cu film bridge; the duration time is 250 μs. While for the (Al/PTFE)_n_/Cu film bridge, a fiercer explosion process with larger quantities of ejected product upwards is observed. The duration time is over 500 μs, which is double that of the Cu film bridge. These results reveal clearly that the chemical reaction of Al/PTFE nanolaminates participates in the ionization of the Cu film bridge, and the energy release of Al/PTFE nanolaminates can improve the electric initiation performances substantially. The potential gas release and larger quantities of ejected product upwards is beneficial for increasing the pressure of generated plasma.

**Fig. 6:**
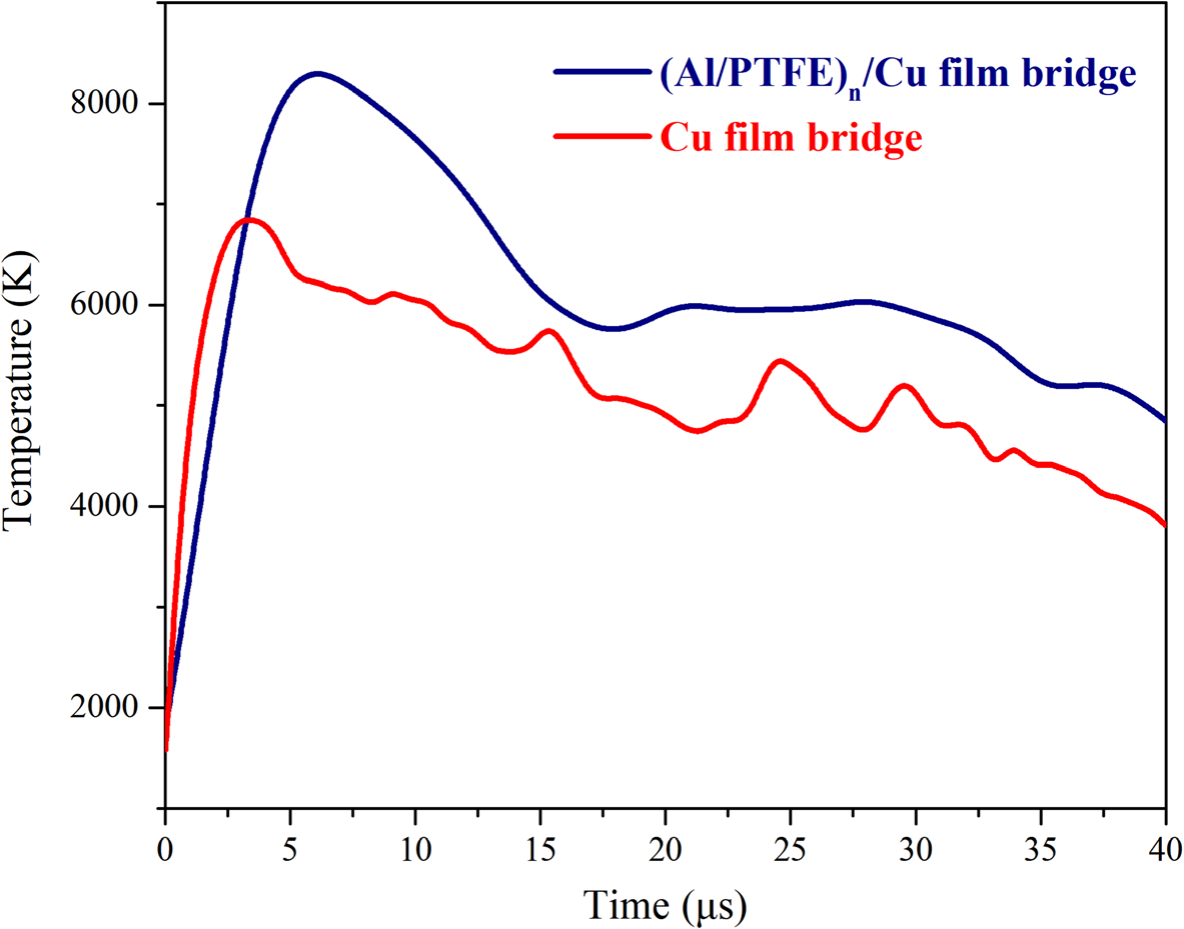
The temperature variation curves after data processing during the electric explosion process for the Cu film bridge and the (Al/PTFE)_n_/Cu film bridge at a 2500-V discharge voltage

It is a difficult work to measure the transient temperature for the electric explosion temperature may reach several thousand degrees Kelvin within micro- or nanoseconds. In this article, the temperature variations of plasma during the initiation processes are determined by comparing relative intensities of spectral lines from the same atomic or ionic species. Figure 6 shows the plasma temperature variations of the Cu film bridge and the (Al/PTFE)_n_/Cu film bridge during the electric initiation processes. After triggering, the electric explosion temperature of the Cu film bridge increases rapidly and arrives at the maximum of ~ 6819 K. While for the (Al/PTFE)_n_/Cu film bridge, the peak temperature is ~ 8289 K; it is much higher than that of the Cu film bridge. It indicates clearly that the chemical reaction in Al/PTFE nanolaminates is triggered with a large number of heat release. The higher temperature is beneficial to the ionization of metal film and the expansion of plasma rapidly. These results are well consistent with the high-speed observation.

**Fig. 7:**
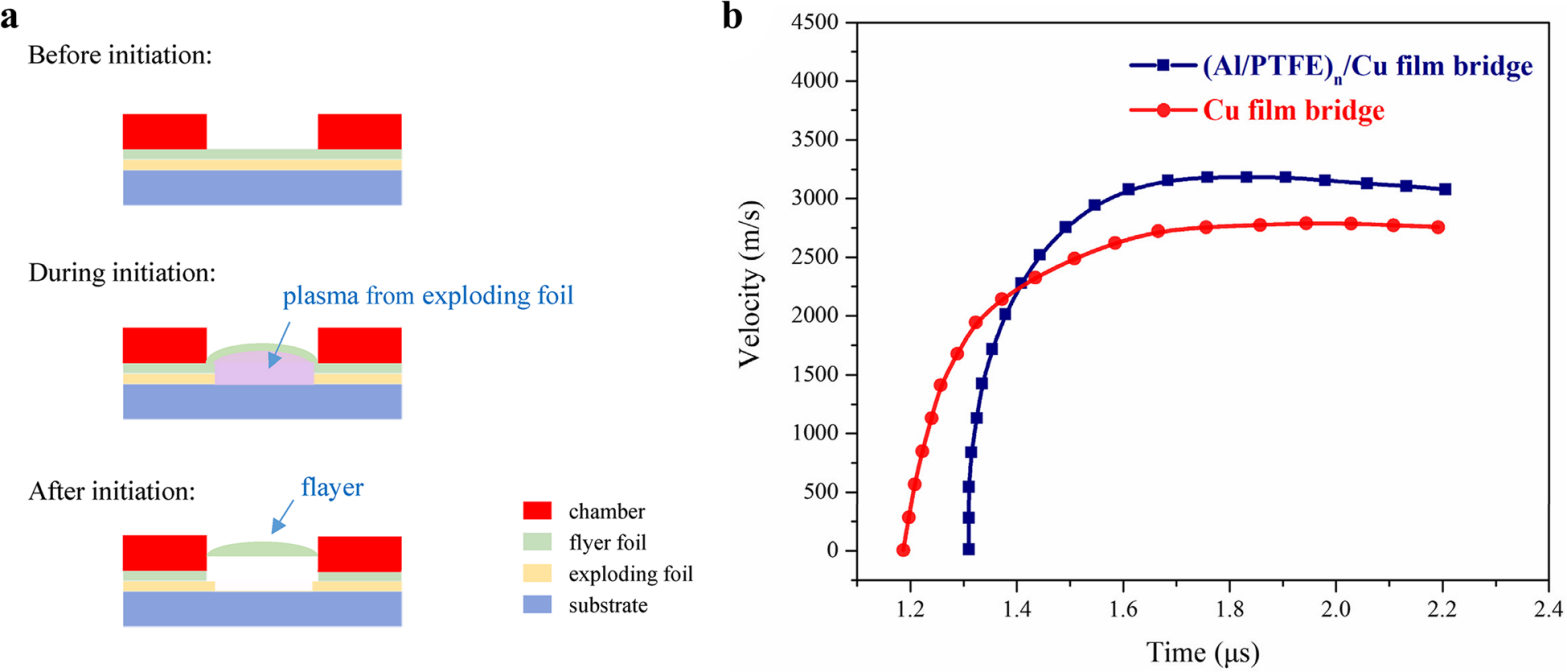
**a** Schematic illustration side view of EFI operation in electric initiation process. **b** The velocity variation curves reconstructed from PDV signal during the electric initiation processes for the Cu film bridge and the (Al/PTFE)_n_/Cu film bridge at a 2500-V discharge voltage

As we all know, the final velocity of flyers will affect the successful detonation of the explosives, and the kinetic energy of flyers derives from the rapid expansion of Cu plasma. After triggering, the insulated flyer foil coated on the film bridge is sheared and pushed upwards by the high temperature and pressure plasma, as shown in Fig. 7a. The velocity variations with time were reconstructed from PDV signal through fast Fourier transformation [29]. Figure 7b shows the velocity variation curves for the Cu film bridge and the (Al/PTFE)_n_/Cu film bridge at a 2500-V discharge voltage. As the bridge film vaporizes and the plasma expands rapidly, the flyer layer begins to form a bubble and then be cut out by the edge of the barrel. The flyer is accelerated upwards until it reaches a balance between air-resistance and pressure from the explosion, and subsequently occurs a platform. The peak velocity is 2792 m/s for the Cu film bridge, while it is 3180 m/s for the (Al/PTFE)_n_/Cu film bridge. These mean that the kinetic energy of flyers derived from the electric explosion is increased around 29.9% owing to the integration with Al/PTFE nanolaminates. Although the launch time for (Al/PTFE)_n_/Cu film bridge is a bit later than that of the Cu film bridge, the overall acceleration time is quite approximate. The chemical reaction of the Al/PTFE nanolaminates is in accord with the electric explosion of Cu film bridge, and the energy output of the Cu film bridge can be increased evidently through integration with the Al/PTFE nanolaminates.

## Conclusions

In summary, reactive Al/PTFE nanolaminates with periodic layer structure were successfully fabricated by magnetron sputtering. The Al/PTFE nanolaminates was composed of PTFE layers (amorphous), Al layers (polycrystalline), and inert layers (Al-F compound) in a metastable system, which could provide a high energy output of 3034 J/g. Through MEMS technology, the Al/PTFE nanolaminates were integrated with a Cu exploding foil to construct an integrated film bridge. The chemical reaction of Al/PTFE nanolaminates is well consistent with the electric explosion of the Cu film bridge. The electric explosion temperature and the energy output of the integrated film bridge are also increased evidently. Overall, the initiation performances of the Cu film bridge can be improved obviously through integration with the Al/PTFE nanolaminates.

## Abbreviations

Al: Aluminum
Cu: Cuprum
DSC: Differential scanning calorimetry
EFI: Exploding foil initiator
MEMS: Microelectronic and mechanical systems
NOC: Nanoenergetic-on-a-chip
PTFE: Polytetrafluoroethylene
TEM: Transmission electron microscopy
XPS: X-ray photoelectron spectroscopy

## References

[1] Sundaram D, Yang V, Yetter RA (2017). Metal-based nanoenergetic materials: synthesis, properties, and applications. Prog Energy Combust Sci.

[2] Zhou X, Xu D, Lu J, Zhang K (2015). CuO/Mg/fluorocarbon sandwich-structure superhydrophobic nanoenergetic composite with anti-humidity property. Chem Eng J.

[3] Adams DP (2015). Reactive multilayers fabricated by vapor deposition: a critical review. Thin Solid Films.

[4] Kim SB, Kim KJ, Cho MH (2016). Micro- and nanoscale energetic materials as effective heat energy sources for enhanced gas generators. ACS Appl Mater Interfaces.

[5] Rogachev AS, Vadchenko SG, Baras F (2016). Combustion in reactive multilayer Ni/Al nanofoils: experiments and molecular dynamic simulation. Combust Flame.

[6] Glavier L, Taton G, Ducéré JM (2015). Nanoenergetics as pressure generator for nontoxic impact primers: comparison of Al/Bi_2_O_3_, Al/CuO, Al/MoO_3_ nanothermites and Al/PTFE. Combust Flame.

[7] Manukyan KV, Tan W, Deboer RJ (2015). Irradiation-enhanced reactivity of multilayer Al/Ni nanomaterials. ACS Appl Mater Interfaces.

[8] Marín L, Nanayakkara CE, Veyan JF (2015). Enhancing the reactivity of Al/CuO nanolaminates by Cu incorporation at the interfaces. ACS Appl Mater Interfaces.

[9] Rogachev AS, Vadchenko SG, Baras F (2014). Structure evolution and reaction mechanism in the Ni/Al reactive multilayer nanofoils. Acta Mater.

[10] Becker CR, Apperson S, Morris CJ (2011). Galvanic porous silicon composites for high-velocity nanoenergetics. Nano Lett.

[11] Tanaka S, Kondo K, Habu H (2008). Test of B/Ti multilayer reactive igniters for a micro solid rocket array thruster. Sensors Actuators A Phys.

[12] Wang J, Besnoin E, Knio OM, Weihs TP (2004). Investigating the effect of applied pressure on reactive multilayer foil joining. Acta Mater.

[13] Baginski TA, Taliaferro SL, Fahey WD (2015). Novel electroexplosive device incorporating a reactive laminated metallic bridge. J Propuls Power.

[14] Rossi C, Zhang K, Estève D (2007). Nanoenergetic materials for MEMS: a review. J Microelectromech Syst.

[15] Zhou X, Torabi M, Lu J (2014). Nanostructured energetic composites: synthesis, ignition/combustion modeling, and applications. ACS Appl Mater Interfaces.

[16] Yan YC, Shi W, Jiang HC (2015). Characteristics of the energetic igniters through integrating Al/NiO nanolaminates on Cr film bridge. Nanoscale Res Lett.

[17] Li X, Guerieri P, Zhou W (2015). Direct deposit laminate nanocomposites with enhanced propellent properties. ACS Appl Mater Interfaces.

[18] Zhang Y, Jiang H, Xing D (2017). B/Ti nano-multilayers as effective heat energy source for enhanced micro-initiator. Appl Therm Eng.

[19] Xu D, Yang Y, Cheng H (2012). Integration of nano-Al with Co_3_O_4_ nanorods to realize high-exothermic core-shell nanoenergetic materials on a silicon substrate. Combust Flame.

[20] Davies H, Chapman DJ, Vine TA, Proud WG (2009) Characterisation of an exploding foil initiator (EFI) system. Aps Topical Conference on Shock Compression of Condensed Matter, American Institute of Physics, pp 283–286

[21] Fischer SH, Grubelich MC (1998). Theoretical energy release of thermites, intermetallics, and combustible metals.

[22] Williams RA, Patel JV, Ermoline A (2013). Correlation of optical emission and pressure generated upon ignition of fully-dense nanocomposite thermite powders. Combust Flame.

[23] Zhou X, Shen R, Ye Y (2011). Influence of Al/CuO reactive multilayer films additives on exploding foil initiator. J Appl Phys.

[24] Zhu P, Shen R, Fiadosenka NN (2011). Dielectric structure pyrotechnic initiator realized by integrating Ti/CuO-based reactive multilayer films. J Appl Phys.

[25] Guo R, Hu Y, Shen R (2012). A micro initiator realized by integrating KNO_3_@CNTs nanoenergetic materials with a Cu microbridge. Chem Eng J.

[26] McGuire GE, Schweitzer GK, Carlson TA (1973). Core electron binding energies in some group IIIA, VB, and VIB compounds. Inorg Chem.

[27] Wang SD, Fung MK, Lai SL (2003). Experimental study of a chemical reaction between LiF and Al. J Appl Phys.

[28] Wang J, Jiang X, Zhang L (2015). Design and fabrication of energetic superlattice like-PTFE/Al with superior performance and application in functional micro-initiator. Nano Energy.

[29] Strand OT, Berzins LV, Goosman DR (2005). Velocimetry using heterodyne techniques. Proc SPIE Int Soc Opt Eng.

